# Relating physical exercise to “lying flat” among Chinese college students: the chain mediation of temporal focus and the sense of meaning in life

**DOI:** 10.3389/fpsyg.2026.1681347

**Published:** 2026-02-20

**Authors:** Xueyuan Cao, Yaogang Han

**Affiliations:** School of Physical Education, Shanghai University of Sport, Shanghai, China

**Keywords:** lying flat, mediation, physical exercise, sense of meaning in life, temporal focus

## Abstract

In recent years, the phenomenon of “neijuan” (involution) has intensified pressures among Chinese college students and often leads to burnout and “lying flat”, a state of disengagement characterized by reduced effort and low ambition. We hypothesized that physical exercise might mitigate “lying flat” tendencies, with temporal focus and sense of meaning in life acting as mediators. This study employed a cross-sectional design, surveying 679 undergraduate students. Key results revealed a significant negative correlation between physical exercise and behavioral “lying flat”. Indirect effects were also significant, with the sense of meaning in life mediating the relationship between physical exercise and both behavioral “lying flat” and psychological “lying flat”. Additionally, a chain mediation effect was observed, where temporal focus and sense of meaning in life together mediated the relationship between physical exercise and behavioral “lying flat” as well as psychological “lying flat”. Physical exercise is negatively associated with “lying flat”, particularly behavioral “lying flat”, with temporal focus and sense of meaning in life as chain mediators. These findings indicate the necessity of integrating physical exercise programs and meaning-focused initiatives in universities to combat “lying flat”, potentially improving student well-being and societal outcomes.

## 1 Introduction

In recent years, the term “neijuan” (involution) has gained widespread attention in China, representing an irrational and inefficient competition for scarce, high-quality resources (Yi et al., 2022). This phenomenon is deeply entrenched in China's exam-oriented education system, where the high-stakes Gaokao (China's National College Entrance Exam) and societal emphasis on extrinsic motivations, such as prestigious university admissions, parental approval, and future economic stability, drive students into a relentless cycle of overwork and diminishing returns (Meng-ying, 2021). Rooted in Confucian ideals of diligence and meritocracy, neijuan reflects broader East Asian competitive cultures, demonstrated in South Korea's “education fever” and Taiwan's intense tutoring industries, where extrinsic rewards overshadow intrinsic learning and contribute to widespread burnout burnout (Koo, 2016). This phenomenon is particularly pronounced among Chinese college students, who face intense academic pressures to secure better grades, internships, and future career opportunities in a highly competitive environment. The relentless pursuit of academic excellence often leads to burnout, stress, and a sense of futility. These negative affective factors prompt many students to adopt a counter-cultural response known as “lying flat” (Dong, 2022).

### 1.1 “Lying flat”

“Lying flat” became the 2021 annual hot word on the Internet in China (Ou, 2023), and has attracted extensive scholarly attention since then. Often termed “tangping” in Chinese, “lying flat” embodies a form of passive resistance to neijuan, drawing from traditional East Asian philosophies such as Taoism's “wu wei” (non-action) and Buddhism's detachment from worldly desires, reinterpreted in contemporary contexts to advocate for minimalism, low ambition, and withdrawal from societal pressures (Zhang et al., 2025). Critically, while it offers a critique of extrinsic-driven cultures by prioritizing mental health over endless competition, it risks fostering societal stagnation, such as reduced innovation and demographic challenges like low birth rates. Parallels can be drawn to Japan's “low-desire society” or South Korea's “Sampo generation” (Lu et al., 2025; Hsu, 2022). It features various meanings and connotations. “Lying flat” can be a state in which people choose to give up their efforts and passively escape when the pressure exceeds their psychological threshold (Xiong, 2022). It can also be an active choice aiming at adjusting oneself and temporarily relaxing (Zhou et al., 2024). “Lying flat” includes both a psychological dimension and a behavioral dimension (Peng, 2023). Psychologically, “lying flat” is the mentality of not competing, having low desires, and feeling satisfied with the status quo. Behaviorally, “lying flat” refers to avoidance of life pressure and pursuit of only few material needs.

“Lying flat” is a global phenomenon (Dai, 2022), however, its prevalence in China underscores the unique intersection of rapid economic growth, educational involution, and cultural expectations, making it a lens for examining youth disengagement in China. The phenomenon of “lying flat” among young people is not only present in China, but also in other countries, such as the “NEET” in South Africa and some European countries, the “low desire society” in Japan, and the “solitary society” in South Korea. For college students, choosing to “lie flat” in early adulthood is not conducive to realizing one's self-worth and may ultimately lead to a decline in life quality (Song and Bie, 2022). If “lying flat” spreads among young people, it will give rise to “reclusive mentality, pessimism, celibacy, and infertility” (Dong, 2022), which can negatively impact the whole society. Therefore, it is necessary and urgent to investigate “lying flat” among the youth group.

Previous research has classified “lying flat” from various perspectives, such as active or passive “lying flat” (Song and Bie, 2022). Classification based on reasons for “lying flat” has attracted much scholarly attention (Qin and Dai, 2022). The first is “escaping-style lying flat” as an active choice to avoid earthly responsibilities. The second is “helpless-style lying flat” as a passive choice after making desperate efforts. The third is “self-deprecating-style lying flat”, which means “lying flat” in mentality but not giving up on striving in action. People of this type mainly express dissatisfaction and rebellion toward “involution” to relieve pressure and adjust mentality (Qin and Dai, 2022). These classifications, however, often fail to account for cases where individuals exhibit behavioral “lying flat” without the “lying-flat” mindset, a state that may combine expectation for better life but without action and represent the most distressing form of “lying flat”. Existing research has not distinctly separated the psychological and behavioral dimensions in measurement, a gap this study seeks to address. From both psychological and behavioral perspectives, “lying flat” may include three categories: “psychologically-only lying flat”, “behaviorally-only lying flat”, and “completely lying flat”.

“Lying flat” measurement is not yet satisfactory. Some studies even use only two questions for measurement: “Are you lying flat now” and “Have you been lying flat for a long time” (Yin and Zhou, 2024). Lu noticed that lack of appropriate measurement tools confines “lying flat” research to theoretical discussion, so they developed the “Lying Flat” Tendency Scale. This scale has the advantages of stable single dimension and concise structure (6 items), and has been used in many studies (Lu et al., 2024; Zhu et al., 2025). Additionally, some scales can measure “lying flat” characteristics. The avoidance behavior in “lying flat” can be measured by the Cognitive Behavioral Avoidance Scale (Ottenbreit and Dobson, 2004), burnout behavior by the Maslach Burnout Inventory (Maslach and Jackson, 1981), and low desire mentality by the Material Values Scale (Richins and Dawson, 1992).

“Lying flat” may be caused by stress. According to the “self-determination theory”, enormous social stress leads to dissatisfaction in basic psychological needs (e.g., autonomy, competency, and connection) among some young people, and thus greatly reduces their achievement motivation. Ultimately, they may choose to “lie flat” (Zhu and Guan, 2023). As such, “lying flat” is caused by low intention to self-improvement (Zheng et al., 2023). Another cause is stress from employment, life, and peer competition (Lin and Gao, 2021; Zhu and Wang, 2018). Stress can be well alleviated by physical exercise (Klaperski and Fuchs, 2021; Tao et al., 2023; Yoon et al., 2023). Therefore, physical exercise may help with lower levels of “lying flat” through its potential link to reduced stress. In this sense, the present study puts forward Hypothesis 1: Physical exercise is negatively associated with the degree of “lying flat” among college students.

### 1.2 The sense of meaning in life and its relationship with “lying flat”

The sense of meaning in life is the core motivation of living as a human being (King and Hicks, 2021). This concept refers to the degree to which an individual understands and comprehends the meaning of life, accompanied by their awareness of the purpose, mission, and primary goal of life (Steger et al., 2006). To measure the sense of meaning in life, Steger et al. (2006) compiled a questionnaire, which divided this concept into two dimensions: the existence of meaning and the search for meaning. This scale has been widely adopted in previous research (Huang et al., 2023; Martela and Steger, 2016; Yek et al., 2017).

One's sense of meaning in life is closely related to their physical and mental health (Pan, 2020). With appropriate sense of meaning in their lives, difficulties and stressors in daily life can be seen as challenges rather than threats to life experiences (Park and Baumeister, 2017). A stronger sense of meaning in life is associated with lower individual stress (Shin, 2022). Based on the relationship between stress and “lying flat” mentioned earlier, we can infer that the sense of meaning in life may also be related to “lying flat”. Besides, the confusion and increased stress associated with a vague sense of meaning in life are linked to “lying flat” (Lin and Gao, 2021; Zhang et al., 2022; Zhu and Wang, 2018). People who lack a sense of meaning in life are more likely to choose “lying flat”. Thus, this study proposes Hypothesis 2: The sense of meaning in life is negatively associated with the degree of “lying flat” among college students.

### 1.3 Temporal focus and its relationship with the sense of meaning in life and “lying flat”

Human has never stopped exploration of time has never stopped. Time can be divided into two categories: objective time and subjective time. The former is the absolute time measured by clocks or calendars (Zerubavel, 1985), while the latter is the individual and collective perception of time (Levasseur et al., 2020). An important concept concerning subjective time is temporal focus. Temporal focus refers to individuals attention to thinking about the time, reflecting the importance placed on time, which is considered a component of the time perspective along with time depth, psychological activity, and rhythm preferences (Chishima et al., 2017). A similar concept, time perspective refers to an individual's overall view of their psychological past and future at a specific objective time point (Smith, 1951), reflecting how people perceive and use time. Temporal focus can be measured with Shipp temporal focus scale in terms of the degree to which an individual focuses their attention on time (Shipp et al., 2009).

Time is the tool people use to give meaning to life and control it (Eryılmaz, 2011). One's temporal focus and their sense of meaning in life is significantly related (Marija and Elena, 2014). For young people, the more inclined they are toward time focus, the stronger their sense of meaning in life (Baikeli et al., 2021). Based on this, this study proposes a Hypothesis 3: Temporal focus is positively associated with the degree of sense of meaning in life among college students. However, to the best of our knowledge, no previous research has examined the impact of temporal focus on “lying flat”. Some studies suggest that individuals with a stronger time focus are more focused on self-improvement and are more likely to proactively set goals and take actions (Ferrari and Díaz-Morales, 2007; Seijts, 1998). Self-improvement is one of the important factors associated with “lying flat” (Zheng et al., 2023). Therefore, temporal focus may have an impact on “lying flat”. Thus, Hypothesis 4 is proposed in this study: Temporal focus is negatively associated with the degree of “lying flat” among college students.

### 1.4 Physical exercise and its relationship with temporal focus, the sense of meaning in life, and “lying flat”

Physical exercise refers to any physical activity that promotes physical and psychological development, with a certain intensity, frequency, and duration (CM, 2009; Morgan and Ellickson, 2021). The physical exercise rating scale by Deqing (1994) evaluates physical exercise from three aspects: intensity, frequency, and duration, with high credibility, widely used in previous research. Moderate physical exercise is beneficial to physical and psychological health (Curran et al., 2020; Ozemek et al., 2018). The impact of physical exercise on individual mental health has attracted extensive attention (Feng et al., 2024; Liu et al., 2024).

Physical exercise is closely related to sense of meaning in life, temporal focus and “lying flat”. Zhao and Yin (2024) found a significantly positive correlation between physical exercise and college students' sense of meaning in life scores, indicating that engaging in physical exercise is associated with higher sense of meaning in life. Relatively little research has investigated the relationship between physical exercise and temporal focus, but individuals with high scores in physical exercise are more likely to make plans for future (Nguyen, 2016). In this sense, this study puts forward Hypothesis 5: physical exercise is positively associated with sense of meaning in life among college students, Hypothesis 6: physical exercise is positively related to the degree of temporal focus among college students, Hypothesis 7: physical exercise can indirectly predict college students “lying flat” through the sense of meaning, and Hypothesis 8: physical exercise is indirectly associated with “lying flat” of college students through temporal focus.

Based on the eight hypotheses, this study proposes Hypothesis 9: physical exercise is associated with “lying flat” of college students indirectly through chain mediation of temporal focus and the sense of meaning in life.

**Figure 1:**
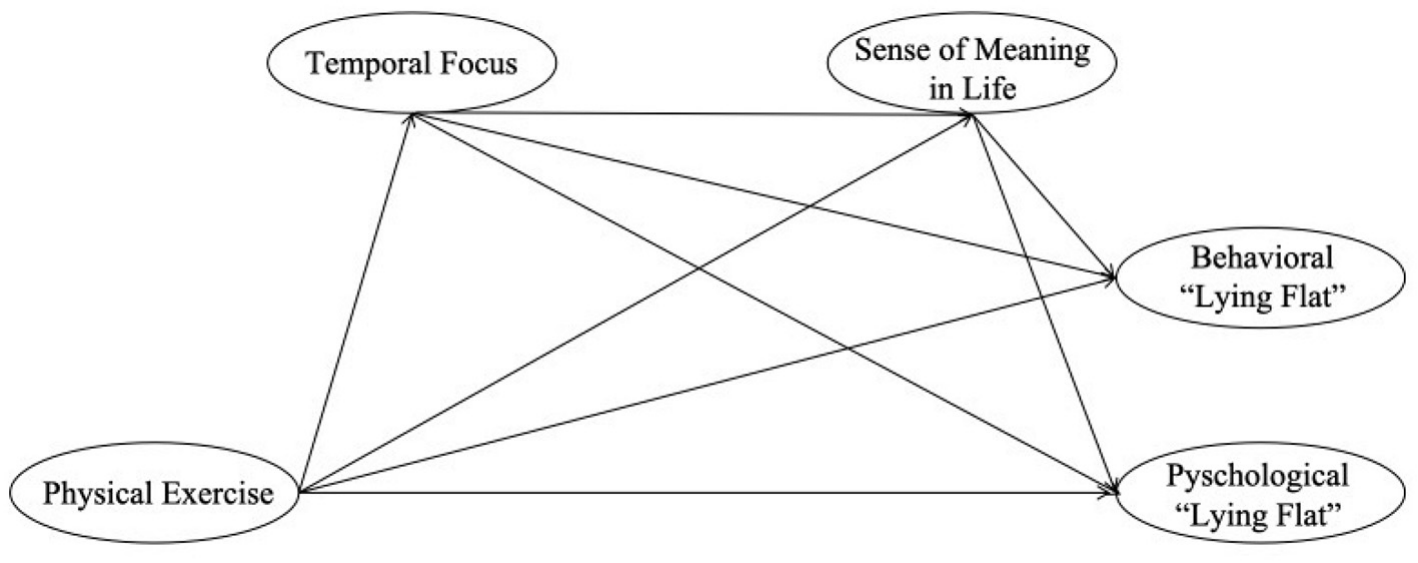
Hypothesized conceptual model.

This study examines the association between physical exercise and the “lying flat” phenomenon among Chinese college students, elucidating the chain mediating roles of temporal focus and sense of meaning in life. It investigates the associations between physical exercise and “lying flat”, with temporal focus and sense of meaning in life as potential mediators, offering insights into psychological intervention mechanisms. Theoretically, it advances the understanding of “lying flat” by integrating physical exercise with temporal focus and sense of meaning in life constructs, contributing a novel framework to psychological research. Practically, the findings can inform university mental health and physical education strategies to mitigate “lying flat” and enhance students wellbeing. Moreover, it provides empirical evidence to support interventions addressing youth disengagement, therefore, fostering societal positivity. The model diagram is shown in Figure 1.

### 1.5 Gender differences in physical exercise, temporal focus, sense of meaning in life, and “lying flat”

Gender differences have been consistently documented across the key constructs, which may moderate the proposed chain mediation model. Chinese female college students tend to report lower levels of moderate-to-vigorous physical activity and higher sedentary time than males, while also deriving greater psychological benefits (e.g., reduced depression, enhanced self-control) from the same amount of exercise (Bai et al., 2025). With respect to temporal focus, females generally exhibit higher sensitivity to time passage and are more likely to adopt a present-fatalistic or past-negative orientation under stress (Płaszowa et al., 2025). Regarding sense of meaning in life, women in emerging adulthood typically report a stronger active search for meaning and higher relational sources of meaning, whereas men emphasize achievement and self-transcendence (Karayigit et al., 2025). Finally, female college students in China display higher academic burnout, emotional exhaustion, and perceived stress—key antecedents of “lying flat”—compared to males (Karaman et al., 2019). These gender differences suggest that the strength of the indirect pathways from physical exercise → temporal focus → sense of meaning in life → “lying flat” may vary between males and females. Therefore, this study proposes an additional exploratory hypothesis: Hypothesis 10: The chain mediating effects of temporal focus and sense of meaning in life in the relationship between physical exercise and both behavioral and psychological “lying flat” differ significantly between male and female college students.

## 2 Methods

### 2.1 Overall research design

This study employs a cross-sectional research design to investigate the relationship between physical exercise and “lying flat”, while also considering temporal focus and the sense of meaning in life as chain mediators.

### 2.2 Research settings and participants

This study used convenience sampling, and a questionnaire survey was administered in April 2025 among undergraduate students (freshmen to seniors). Different types of universities (comprehensive university, finance and economics university, science and technology university and sports university) were chosen to guarantee representativeness of the sample. Additionally, the sample included participants from various majors, grade levels and genders, further ensuring the diversity and representativeness of the sample. We distributed the questionnaire through the Wenjuanxing platform, an online questionnaire tool. To ensure the completeness and validity of responses, all items in the questionnaire were set as mandatory, requiring respondents to complete all items before submitting the questionnaire. Through this method, we successfully collected data without any missing values. All research methods involving human participants in this study were conducted in strict accordance with relevant guidelines and regulations. Consents were gained from all participants. A total of 726 responses were collected, of which 679 were valid after excluding irregular responses, yielding an effective response rate of 93.5%.

As shown in Table 1, all the 679 participants were undergraduate students, including 384 males and 295 females, aged between 17 and 22 years old. They are mainly freshmen (407) and sophomores (247). Their majors cover different fields such as electrical engineering, automation, big data, law, economics, business management, and physical education.

**Table 1:** Background information of participants.

**Grade**	**Male**	**Female**	**Total/proportion**
Freshman	257	150	407/0.5994
Sophomore	118	129	247/0.3638
Junior	45	106	14/0.206
Senior	5	6	11/0.0162
Total/proportion	384	295	679/1.0

### 2.3 Variables and measurements

#### 2.3.1 Physical exercise

The Physical Exercise Scale with six items was designed to assess participants' engagement in physical activities, capturing the quantity and subjective experience of exercise. The quantity section incorporated the Physical Activity Rating Scale (PARS-3) adapted from Deqing (1994), to measure the quantity of physical activity based on intensity, frequency, and time (combined together to be the quantity of physical exercise = intensity × (time-1) × frequency). Additionally, items from the Exercise Identity Scale (Anderson and Cychosz, 1994) and the Intrinsic Motivation for Sport Scale (Pelletier et al., 1995) were included to assess subjective experiences of physical exercise, rated on a 5-point Likert scale (1 = strongly disagree, 5 = strongly agree). Example items include: “I have many goals related to exercising” (from Exercise Identity Scale) and “I participate in physical exercise because it is fun” (from Intrinsic Motivation Inventory, interest/enjoyment subscale). The scale exhibited excellent reliability, with Cronbach's α = 0.889, composite reliability (CR) = 0.903, and average variance extracted (AVE) = 0.620. To assess the appropriateness of the data for factor analysis, the Kaiser-Meyer-Olkin (KMO) Measure of Sampling Adequacy and Bartlett's Test of Sphericity were conducted. The KMO value was 0.865, indicating a high degree of sampling adequacy, and Bartlett's test was significant (*x*^2^ = 2515.217, df = 10, *p* < 0.001), supporting the application of factor analysis. Confirmatory Factor Analysis (CFA) was conducted to validate the three-factor structure of the scale. The model demonstrated acceptable fit: *x*^2^/df = 2.874, *p* < 0.001, GFI = 0.991, TLI = 0.993, SRMR = 0.020, RMSEA = 0.052 (90% CI: [0.021, 0.085]), as shown in Table 2.

**Table 2:** Results of confirmatory factor analysis for the subscales and the overall scale.

**Variable**	***x*^2^/df**	**RMSEA**	**GFI**	**TLI**	**SRMR**	**Cronbach's α**	**KMO**	**AVE**	**CR**
PE	2.847	0.052	0.991	0.993	0.02	0.889	0.865	0.62	0.903
SML	2.918	0.053	0.993	0.993	0.009	0.93	0.897	0.722	0.928
TF	1.697	0.032	0.99	0.994	0.012	0.87	0.817	0.554	0.859
BLF	2.633	0.049	0.984	0.983	0.024	0.882	0.886	0.637	0.875
PLF	2.633	0.049	0.984	0.983	0.024	0.743	0.886	0.57	0.728

PE, Physical Exercise; SML, Sense of Meaning in Life; TF, Temporal Focus; BLF, Behavioral “Lying Flat”; PLF, Psychological “Lying Flat”.

#### 2.3.2 Temporal focus

The Temporal Perspective Scale was adapted from the time perspective measures developed by Shipp et al. (2009), designed to assess participants temporal focus. The scale comprised eight items, rated on a 5-point Likert scale (1 = strongly disagree, 5 = strongly agree), with higher scores indicating greater attention to time. Example items include: “I think back to experiences I have had”, “I focus on what is happening to me right now”, and “I think about what my future has in store”. The scale showed good reliability, with Cronbach's α = 0.930, CR = 0.928, and AVE = 0.722. The KMO value was 0.817, indicating a high degree of sampling adequacy, and Bartlett's test was significant (*x*^2^ = 3200.999, df = 28, *p* < 0.001), supporting the application of factor analysis. The model demonstrated acceptable fit: *x*^2^/df = 1.697, *p* < 0.001, GFI = 0.990, TLI = 0.994, SRMR = 0.012, RMSEA = 0.032 (90% CI: [0.008, 0.052]), as shown in Table 2.

#### 2.3.3 The sense of meaning in life

The Sense of Meaning in Life Scale was adapted from the Meaning in Life Questionnaire compiled by Steger et al. (2006), utilizing the “presence of meaning” dimension to assess the extent to which participants perceived their lives as meaningful (e.g., “I understand my life's meaning” and “My life has a clear sense of purpose”). The scale consisted of five items, rated on a 5-point Likert scale (1 = strongly disagree, 5 = strongly agree), with higher scores indicating a clearer sense of meaning in life. The scale exhibited excellent reliability, ores indicating a clearer sense of meaning in life. The scale exhibited excellent reliability, with Cronbach's α = 0.870, CR = 0.859, and AVE = 0.554. The KMO value was 0.897, indicating a high degree of sampling adequacy, and Bartlett's test was significant (*x*^2^ = 2671.468, df = 10, *p* < 0.001), supporting the application of factor analysis. The model demonstrated acceptable fit: *x*^2^/df = 2.910, *p* < 0.001, GFI = 0.993, TLI = 0.993, SRMR = 0.009, RMSEA = 0.053 (90% CI: [0.019, 0.090]), as shown in Table 2.

#### 2.3.4 “Lying flat”

The “Lying Flat” Scale was developed to assess the behavioral and psychological dimensions of the “lying flat” phenomenon, drawing on the “Lying Flat” Tendency Scale by Lu et al. (2024), the Cognitive-Behavioral Avoidance Scale (Ottenbreit and Dobson, 2004), Maslach Burnout Inventory (Maslach and Jackson, 1981), and Material Values Scale (Richins and Dawson, 1992). The scale comprised eight items, with four items per dimension, rated on a 5-point Likert scale (1 = strongly disagree, 5 = strongly agree), with higher scores indicating a greater degree of lying flat tendencies. Example items include: “I don't make an effort to do anything” (behavioral dimension, from Lying Flat Tendency Scale), “I feel emotionally drained from my work” (behavioral dimension, from Maslach Burnout Inventory), and “I have no pursuit of more affluent material conditions” (psychological dimension, adapted reverse from Material Values Scale). The overall scale had a Cronbach's α of 0.874, with behavioral subscale showing Cronbach's α = 0.882, CR = 0.875, AVE = 0.637 and psychological subscale showing Cronbach's α = 0.743, CR = 0.728, AVE = 0.570. The KMO value of the overall scale was 0.886, indicating a high degree of sampling adequacy, and Bartlett's test was significant (*x*^2^ = 2687.855, df = 28, *p* < 0.001), supporting the application of factor analysis. The model demonstrated acceptable fit: *x*^2^/df = 2.633, *p* < 0.001, GFI = 0.984, TLI = 0.983, SRMR = 0.024, RMSEA =0.049 (90% CI: [0.032, 0.067]), as shown in Table 2.

#### 2.3.5 Sample size estimation

To decide adequate sample size for the proposed structural equation model (SEM), an a priori sample size estimation was conducted using multiple SEM-oriented power analysis tools. First, Soper's Structural Equation Model Sample Size Calculator was applied using the actual model characteristics extracted from the AMOS output. Assuming a medium anticipated effect size (λ = 0.30), α = 0.05, and desired power of 0.80, the calculator indicated that the minimum required sample size for this level of model complexity is approximately N ≈ 450–500. To verify these results, an additional Monte Carlo simulation-based power analysis was performed using Mplus. Based on 1,000 replications with the same model structure and expected effect sizes, the simulation showed stable parameter recovery and statistical power above 0.80 across all focal direct and indirect paths, suggesting that a sample larger than 500 is sufficient for reliable estimation of chain mediating effects.

Besides, the researchers also adhered to the recommendations by Hair et al. (2019): a minimum of 300 participants is required when the model includes up to 7 constructs, while a minimum of 500 is needed if the number of constructs exceeds seven. The sample size of this study (679) meets this requirement.

#### 2.3.6 Statistical methods

The raw data in Excel format was imported into SPSS 29.0 for descriptive statistics and reliability analysis, normal distribution test (skewness and kurtosis indices), Harman's single-factor test to detect common method bias, and Pearson correlation analyses to analyze the relationships between key variables. Amos 26.0 was used to conduct confirmatory factor analysis, chained mediation effect analysis with a Bootstrapping method (5,000 resamples).

## 3 Results

### 3.1 Common method bias test

The variables in this study were derived from self-reported questionnaire data, necessitating a test for common method bias. The Harman's single-factor test results showed that 19 factors had eigenvalues greater than 1, with the largest factor accounting for 32.96% of the variance, which is below the critical threshold of 40%. Therefore, it can be inferred that there is no significant common method bias in the data, meeting the requirements for statistical analysis.

### 3.2 Descriptive statistics and correlations

Descriptive statistics are shown in Table 3. Normality criteria were met across all variables, as demonstrated by absolute skewness indices (range: −3 to 3) and kurtosis coefficients (range: −10 to 10) (Kline, 2008), thereby supporting subsequent parametric analyses.

**Table 3:** Statistics of descriptive and pearson correlation coefficient.

**Variable**	**M±SD**	**Skewness**	**Kurtosis**	**1 (PE)**	**2 (TF)**	**3 (SML)**	**4 (BLF)**	**5 (PLF)**
1.PE	3.00 ± 0.86	−0.366	0.001	–				
2.TF	3.91 ± 0.57	−1.262	5.758	0.407**	–			
3.SML	3.50 ± 0.86	−0.411	0.243	0.444**	0.550**	–		
4.BLF	2.00 ± 0.77	0.807	1.163	−0.282**	−0.210**	−0.263**	–	
5.PLF	2.45 ± 0.79	0.290	0.085	−0.270**	−0.178**	−0.253**	0.666**	–

^*^p < 0.05, ^**^p < 0.01, ^***^p < 0.001.

Pearson correlation analysis was utilized to assess the pairwise associations among physical exercise, temporal focus, sense of meaning in life, and both behavioral and psychological dimensions of the “lying flat”, as shown in Table 3. Physical exercise demonstrated significant negative correlations with behavioral “lying flat” (r = −0.282, *p* < 0.001) and psychological “lying flat” (r = −0.270, *p* < 0.001). The sense of meaning in life negatively correlated with behavioral (r = −0.263, *p* < 0.001) and psychological “lying flat” (r = −0.253, *p* < 0.001). Temporal focus was positively associated with the sense of meaning in life (r = 0.550, *p* < 0.001), while showing negative relationships with behavioral (r = −0.210, *p* < 0.001) and psychological “lying flat” (r = −0.178, *p* < 0.001). The findings indicated a statistically significant positive correlation between physical exercise and temporal focus (r = 0.407, *p* < 0.001), as well as between physical exercise and the sense of meaning in life (r = 0.444, *p* < 0.001). Moreover, a strong positive correlation was observed between the behavioral and psychological aspects of “lying flat” (r = 0.666, *p* < 0.001), suggesting they are closely intertwined and may reflect a unified underlying disposition. The findings provide evidence that the measurement scales exhibit satisfactory discriminant validity.

### 3.3 The chain mediating effects analysis

Based on the structural equation modeling (SEM) analysis, the relationships among physical exercise, temporal focus, sense of meaning in life, behavioral “lying flat”, and psychological “lying flat” were examined. To control for potential confounding effects, gender, grade level, and major were introduced as control variables in the model. The analysis utilized a recursive model, employing maximum likelihood estimation. Model fit indices indicated an acceptable fit, with x^2^/df = 2.689, *p* < 0.001, GFI = 0.913, TLI = 0.947, SRMR = 0.051, RMSEA = 0.050 (90% CI: [0.046, 0.054]), as presented in Table 4.

**Table 4:** The results of the model fitting test.

**x^2^/df**	**RMSEA**	**GFI**	**TLI**	**SRMR**
2.689	0.050	0.913	0.947	0.051

As shown in Table 5, physical exercise shows direct negative association with behavioral “lying flat” (β = −0.258, 95% CI [−0.413, −0.104]), but its association with psychological “lying flat” was not statistically significant (β = −0.160, 95% CI [−0.346, 0.026]), partially supporting Hypothesis 1. The relationships of temporal focus with behavioral “lying flat” (β = −0.076, *p* = 0.152) and with psychological “lying flat” (β = −0.062, *p* = 0.249) were not statistically significant, rejecting Hypothesis 4. The sense of meaning in life significantly and negatively predicted both behavioral “lying flat” (β = −0.351, *p* < 0.001) and psychological “lying flat” (β = −0.206, *p* = 0.002), supporting Hypothesis 2. Additionally, physical exercise significantly and positively predicted temporal focus (β = 0.457, *p* < 0.001) (supporting Hypothesis 6) and the sense of meaning in life (β = 0.246, *p* < 0.001) (supporting Hypothesis 5). Temporal focus significantly predicted sense of meaning in life (β = 0.433, *p* < 0.001), supporting Hypothesis 3.

**Table 5:** Chain mediation effects of temporal focus and sense of meaning in life on the relationship between physical exercise and “lying flat”.

**Path**	**Effect size**	**BootSE**	**Boot95%CI**	
			**Lower**	**Upper**
Total effect of PE → BLF	−0.460	0.075	−0.608	−0.315
Total effect of PE → PLF	−0.518	0.089	−0.694	−0.346
PE → BLF	−0.258	0.079	−0.413	−0.104
PE → PLF	−0.160	0.095	−0.346	0.026
Total mediating effect of PE → BLF	−0.202	0.058	−0.317	−0.091
Total mediating effect of PE → PLF	−0.358	0.071	−0.501	−0.222
PE → TF → BLF	−0.056	0.041	−0.142	0.021
PE → SML → BLF	−0.081	0.032	−0.147	−0.022
PE → TF → SML → BLF	−0.065	0.024	−0.115	−0.024
PE → TF → PLF	−0.055	0.048	−0.154	0.036
PE → SML → PLF	−0.168	0.045	−0.259	−0.084
PE → TF → SML → PLF	−0.135	0.038	−0.213	−0.066

PE, Physical Exercise; SML, Sense of Meaning in Life; TF, Temporal Focus; BLF, Behavioral “Lying Flat”; PLF, Psychological “Lying Flat”.

The mediation was tested using the bias-corrected percentile Bootstrap method with 5,000 iterations. The sense of meaning in life significantly mediated the relationship between physical exercise and behavioral “lying flat” (β = −0.081, 95% CI [−0.147, −0.022]) as well as psychological “lying flat” (β = −0.168, 95% CI [−0.259, −0.084]), supporting Hypothesis 7. However, the results indicated non-significant mediating of temporal focus in the relationship between physical exercise and behavioral “lying flat” (β = −0.056, 95% CI [−0.142, 0.021]) and psychological “lying flat” (β = −0.055, 95% CI [−0.154, 0.036]), not supporting Hypothesis 8.

Furthermore, significant chain mediating was observed for temporal focus and sense of meaning in life in the relationship of physical exercise to behavioral “lying flat” (β = −0.065, 95% CI [−0.115, −0.024]) and to psychological “lying flat” (β = −0.135, 95% CI [−0.213, −0.066]), supporting Hypothesis 9. The total effects of physical exercise on behavioral “lying flat” and psychological “lying flat” through temporal focus and sense of meaning in life were −0.460 (95% CI [−0.608, −0.315]) and −0.518 (95% CI [−0.694, −0.346]), respectively.

**Figure 2:**
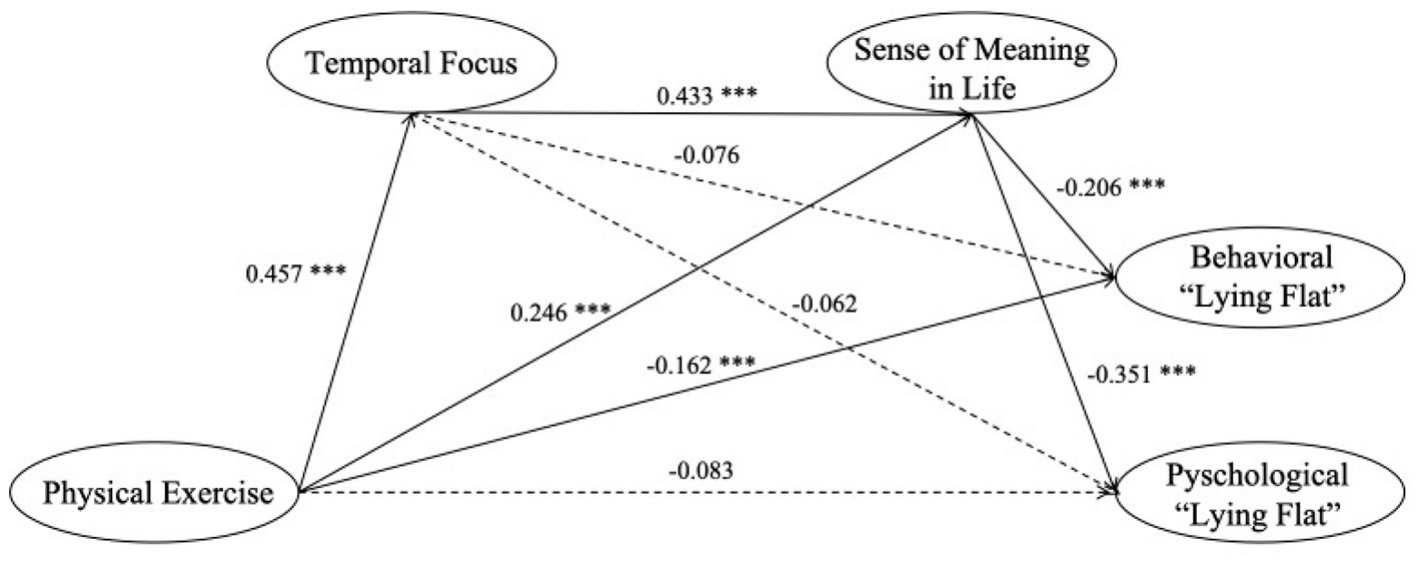
Chain mediation model of temporal focus and sense of meaning in life between physical exercise and “lying flat” (**p < 0.005; ***p < 0.001).

In conclusion, physical exercise exerts direct negative effects on behavioral “lying flat” and indirect negative effects through temporal focus and sense of meaning in life. The results are presented in Table 5 and Figure 2.

### 3.4 Multi-group analyses of the chain mediating effects model according to gender

To examine the applicability of the indirect effects model involving physical exercise, temporal focus, and sense of meaning in life for both genders, multi-group analysis with structural invariance testing was conducted. Separate models were first freely estimated for male students (*n* = 384) and female students (*n* = 295). Excellent fit indices (Table 6) were obtained for the chain mediation model in both groups, confirming the feasibility of conducting multi-group comparisons for the proposed indirect effects model.

**Table 6:** Comparison of indirect effects models.

**Model**	**χ^2^**	**df**	**χ^2^/df**	**GFI**	**AGFI**	**CFI**	**RMSEA**	**Δχ^2^ (Δdf)**	**p**
Male	631.052	283	2.23	0.888	0.86	0.953	0.057	-	-
Fmale	545.756	283	1.928	0.873	0.842	0.943	0.056	-	-
Unconstrained	1,176.836	566	2.079	0.881	0.853	0.949	0.04	-	-
Measurement weights	1,231.017	587	2.097	0.876	0.851	0.947	0.04	54.181(21)	< 0.01
Structural weights	1,265.716	596	2.123	0.873	0.85	0.945	0.041	34.699(9)	< 0.01

PE, Physical Exercise; SML, Sense of Meaning in Life; TF, Temporal Focus; BLF, Behavioral “Lying Flat”; PLF, Psychological “Lying Flat”.

Subsequently, multi-group nested model comparisons were performed across the two gender groups in structural equation modeling (see Table 6). Compared to the unconstrained baseline model (Model 1), imposing measurement weight constraints (Model 3) did not lead to significant deterioration in model fit, Δ*x*^2^(4) = 4.813, *p* = 0.370, indicating that measurement weights were invariant across two gender groups. However, when further imposing structural weight constraints (Model 3) relative to Model 2, model fit deteriorated significantly, Δ*x*^2^(3) = 8.304, *p* = 0.040. These results suggest that, although the measurement model is equivalent between genders, there are significant gender differences in the magnitudes of certain structural paths.

Follow-up pairwise critical ratio tests were conducted to detect differences between groups (|*CR*|≥1.96 considered significantly different). The results revealed several notable gender differences in both total and specific indirect pathways. Regarding the total effects, physical exercise exhibited a significantly stronger negative association with behavioral lying flat among female students (β = −0.428, 95% CI [−0.626, −0.229]) compared with males (β = −0.208, 95% CI [−0.343, −0.087]). A similar trend was found for psychological lying flat, with females again showing a stronger total effect (β = −0.359, 95% CI [−0.376, −0.109]) than males (β = −0.228, 95% CI [−0.376, −0.109]). These results suggest that the role of physical exercise in reducing lying-flat tendencies is more pronounced among female students.

Significant gender differences were also observed in the indirect effects. The total mediating effect of physical exercise on behavioral lying flat was stronger for females (β = −0.198, 95% CI [−0.281, −0.067]) than for males (β = −0.101, 95% CI [−0.156, −0.053]). Similarly, the total mediating effect on psychological lying flat was substantially larger among females (β = −0.293, 95% CI [−0.376, −0.109]) compared with that among males (β = −0.138, 95% CI [−0.201, −0.084]). These findings indicate that the mediating roles of temporal focus and meaning in life are more potent in female students.

Among the specific mediation pathways, pronounced gender differences emerged for the meaning-in-life related routes. For females, both the physical exercise → meaning in life → behavioral lying flat (β = −0.089, 95% CI [−0.173, −0.019]) and physical exercise → meaning in life → psychological lying flat (β = −0.162, 95% CI [−0.277, −0.073]) pathways were statistically significant, whereas these same pathways were non-significant for males (β = −0.004 and β = 0.016, respectively; both 95% CIs included zero). This pattern indicates that meaning in life served as a central mediating mechanism for females but not for males.

For the single mediation through temporal focus, both genders showed significant indirect effects, though females demonstrated slightly larger effect sizes (e.g., physical exercise → temporal focus → behavioral lying flat: females β = −0.078 vs. males β = −0.042; physical exercise → temporal focus → psychological lying flat: females β = −0.075 vs. males β = −0.067). In contrast, males demonstrated stronger effects in the sequential chain mediation pathways. The chain path physical exercise → temporal focus → meaning in life → behavioral lying flat was larger for males (β = −0.055) than for females (β = −0.031), and this difference was even more evident for the chain path predicting psychological lying flat (males β = −0.087 vs. females β = −0.056). These findings indicate that males rely more heavily on the integrated sequence in which improvements in temporal focus enhance meaning in life, ultimately reducing lying fla tendencies.

## 4 Discussion

This study examines the relationship between physical exercise and behavioral and psychological “lying flat” among Chinese college students, with a particular focus on the chain mediation of temporal focus and sense of meaning in life. The findings reveal that physical exercise directly mitigates behavioral “lying flat” but does not directly alleviate psychological “lying flat”. Moreover, physical exercise indirectly influences behavioral and psychological “lying flat” through the independent mediation of temporal focus and sense of meaning in life. Additionally, physical exercise impacts behavioral and psychological “lying flat” via a chain mediation pathway involving temporal focus and sense of meaning in life. This study elucidates the mechanisms through which physical exercise affects behavioral and psychological “lying flat” among college students, providing valuable insights for the prevention and intervention of “lying flat”.

### 4.1 The relationship between physical exercise and “lying flat”

This study shows that there is a significant negative correlation between physical exercise and behavioral “lying flat” in college students. The behavioral “lying flat” is manifested as individuals give up coping with pressure that they cannot bear (Xiong, 2022). This aligns with prior findings that physical exercise alleviates stress and therefore inhibits “lying flat” (Klaperski and Fuchs, 2021; Tao et al., 2023). Regular physical activity may help students maintain active participation in academic and social spheres, counteracting avoidance behaviors associated with behavioral “lying flat”, which may also be the reason why actively participating in physical exercise can inhibit the behavioral “lying flat”.

The direct effect of physical exercise on psychological “lying flat” was not significant. Psychological “lying flat” manifests as satisfaction with the current situation and low desires (Song and Bie, 2022). It suggests that altering the mindset of low ambition or passive contentment requires additional factors and physical exercise may indirectly address the psychological aspects of “lying flat” through mediating variables. Another possibility is that some college students only “lying flat” psychologically without corresponding behavioral manifestations, using psychological “lying flat” as a means of self-regulation in handling difficulties (Zhou et al., 2024).

### 4.2 The mediating effect of temporal focus

The results of temporal focus is consistent with some previous research. Firstly, this study confirms that physical exercise can positively predict temporal focus, and previous studies have also supported this point (Nguyen, 2016). Individuals attending to time are more inclined to set goals, develop plans, and take proactive actions to achieve long-term goals (Lasane and Jones, 1999; Seijts, 1998), which is in line with the characteristics of students that regularly participate in physical exercise (McAuley et al., 2011). In addition, memory and cognitive function are important influencing factors of past temporal focus, and physical exercise significantly enhances these two aspects (Qazi et al., 2024).

However, the impact of temporal focus on behavioral and psychological “lying flat” is not significant, which is inconsistent with the hypothesis. This study defines temporal focus as the degree of attention paid to time. The non-significant direct effect of temporal focus on behavioral and psychological “lying flat” may stem from our unidimensional treatment of temporal focus, which aggregates past, present, and future orientations. As noted, these dimensions can have differential impacts: past focus may heighten stress and rumination (Stolarski et al., 2014), present focus could promote impulsivity (Shipp, 2020),and future focus might enhance motivation and self-improvement (Ferrari and Díaz-Morales, 2007). This oversimplification could mask nuanced effects and explain the lack of a direct relationship, though the chain mediation with sense of meaning in life remains significant. The complex internal mechanism of temporal focus may impact the relationship between temporal focus and “lying flat”. Therefore, the mediating effect of time focus as a whole on physical exercise and “lying flat” was not significant, and more complex mediating effects need to be formed by variables such as the sense of meaning in life to inhibit “lying flat”.

### 4.3 The mediating effect of the sense of meaning in life

The results of sense of meaning in life in this study conforms to all hypotheses. This study proves that physical exercise positively predicts college students sense of meaning in life. This direct relationship may be attributed to exercise-induced positive emotions, such as hope and optimism, which foster a greater sense of purpose in life (Iwon et al., 2021). In addition, physical exercise may also help to foster the sense of meaning in life through other mediating mechanisms, such as self-perception, mindfulness and psychological resilience (Zhao et al., 2024; Jiang and Cao, 2025). The present study found that the sense of meaning in life plays a mediating role in the impact of physical exercise on behavioral and psychological “lying flat”, which is consistent with the hypothesis. Physical exercise is positively related to college students' sense of meaning in life scores (Zhao and Yin, 2024; Zhang et al., 2022). Stronger sense of meaning in life can alleviate individual stress (Shin, 2022)and thus impact “lying flat”. Positive sense of meaning in life can serve as a buffer against stress, which may inhibit “lying flat” (Park and Baumeister, 2017; Zhu and Guan, 2023). Therefore, physical exercise helps individuals gain a deeper understanding of the meaning of life, which in turn suppresses their behavior and psychological “lying flat”.

### 4.4 The chain mediating effect

This study confirms the chain mediating effect of temporal focus and sense of meaning in life between physical exercise and college students' behavior and psychological “lying flat”. This suggests that physical exercise first enhances temporal focus, which subsequently strengthens the sense of meaning in life, ultimately reduces the propensity for “lying flat” (Marija and Elena, 2014). suggest that temporal focus are positively correlated with a greater sense of meaning in life, as they promote goal-directedness and positive reflection on past experiences. The chain mediation can be understood as a sequential psychological pathway. Physical exercise cultivates a temporal focus by improving cognitive clarity and emotional regulation (Qazi et al., 2024). Temporal focus prompts students to envision meaningful life goals, such as academic success or career aspirations, which enhance their sense of purpose (Baikeli et al., 2021). A stronger sense of meaning in life, in turn, mitigates the stress and futility associated with “involution”, discouraging both behavioral withdrawal (e.g., avoiding academic responsibilities) and psychological disengagement (e.g., adopting low desires) (Zhao and Yin, 2024). For instance, a student who regularly jogs may develop a habit of setting fitness goals, which translates into a broader future-oriented mindset. This mindset encourages the student to pursue meaningful academic or extracurricular activities, reinforcing their sense of purpose and reducing the appeal of “lying flat” as a coping mechanism. Therefore, it can be concluded that physical exercise can suppress the behavior and psychological “lying flat” among college students through temporal focus and sense of meaning in life.

### 4.5 Gender differences in the chain mediation mechanism

The multi-group analysis further extends the understanding of the chain mediating relationships by revealing meaningful gender differences in how physical exercise influences both behavioral and psychological lying flat. Overall, these findings underscore that gender plays an important moderating role in the psychological mechanisms underlying the effects of physical exercise on lying-flat tendencies. While female students benefit more directly and strongly from the emotional and existential enhancements associated with physical exercise, males rely more on the sequential interplay between temporal focus and meaning in life. These gender-specific patterns provide valuable guidance for tailoring physical activity and psychological interventions in higher education settings.

First, females exhibited stronger total effects of physical exercise on both behavioral and psychological lying flat compared to males. This suggests that physical exercise may play a more prominent role in buffering disengagement tendencies among female students. One possible explanation is that girls may experience greater stress sensitivity or emotional reactivity, making them more responsive to the mood-regulating and stress-relieving benefits of physical activity (Ordaz and Luna, 2012). As a result, physical exercise may exert a stronger protective effect on reducing avoidance-oriented behaviors and psychological withdrawal among girls.

Second, the results highlighted notable gender asymmetries in the independent mediating role of meaning in life. For females, meaning in life was a central mediator in the relationship between physical exercise and lying flat—both the PE → SML → BLF and PE → SML → PLF pathways were statistically significant. In contrast, these same pathways were non-significant among males. This suggests that female students may be more likely to derive existential benefits, such as enhanced purpose, life clarity, or value orientation, from physical exercise, which subsequently plays a critical role in reducing their lying-flat tendencies. Prior research has suggested that females tend to report higher levels of emotional awareness and introspective processing (Fernández-Berrocal et al., 2012), which may amplify the meaning-enhancing function of physical activity. Thus, interventions aimed at fostering meaning in life may be especially impactful for female students.

Third, although both genders demonstrated significant indirect effects through temporal focus, females again displayed larger effect sizes for the single mediation routes involving temporal focus. This pattern suggests that girls may show a stronger tendency to translate improvements in temporal clarity or time-related attention into reduced disengagement behaviors.

In contrast, males relied more heavily on the chain mediation pathway, the integrated sequence in which physical exercise first enhances temporal focus, which then promotes meaning in life and ultimately reduces lying-flat tendencies. The stronger chain effects observed among males imply that they may require a more structured, sequential psychological process in order to derive benefits from physical exercise. In other words, improvements in meaning in life among males may depend more on prior enhancement in their temporal focus.

### 4.6 Integration of findings with the Chinese and East Asian cultural-educational context

The phenomenon of “lying flat” is not merely a universal form of burnout or disengagement but a culturally embedded collective emotional response deeply rooted in contemporary Chinese society and its high-pressure educational ecosystem. The present findings (physical exercise directly reduces behavioral lying flat, exerts only indirect effects on psychological lying flat, and operates primarily through the chain mediation of temporal focus and sense of meaning in life) are particularly resonant with the high-context, relational, and future-oriented cultural characteristics prevalent in China and most other East Asia countries or regions (Eckhardt, 2002; Markus and Kitayama, 2014).

First, the education systems in the Chinese mainland and many East Asian societies have long revolved around the college entrance examinations and involutionary competition, cultivating a “meaning vacuum” that manifests as psychological lying flat (Xin and Jian, 2024). The chain mediation pathway identified in this study (physical exercise → temporal focus → sense of meaning in life → reduced lying flat) suggests that physical exercise counteracts this tendency precisely by restoring an experiential, controllable, and narrative sense of time, thereby bridging the existential rupture experienced post-Gaokao.

Second, within the enduring influence of Confucian and Daoist traditions in East Asia, low desire and “contentment with the present” are not inherently pathological; Daoist philosophy even valorizes “non-action” (wuwei) and simplicity (D'Ambrosio, 2024). This cultural ambivalence explains why physical exercise alone cannot directly mitigate psychological lying flat: for some students, psychological withdrawal represents a culturally sanctioned form of mental retreat rather than outright malfunction (Sun, 2025). Only when exercise activates an actively constructed, self-endorsed sense of meaning in life rather than externally imposed meaning, can it transform passive contentment into a deliberate choice for a simpler yet purposeful existence.

Finally, the observed gender differences reflect persistent patterns of socialization in contemporary China: female students appear more responsive to the introspective body-emotion-meaning pathway, whereas male students rely predominantly on the structured goal-planning-meaning sequence. These patterns align with traditional and ongoing expectations that emphasize achievement and family establishment for men and emotional regulation for women (Sun et al., 2025).

Thus, beyond confirming the protective role of physical exercise, the current study illuminates the culturally distinctive mechanisms through which this protection operates within China's post-Gaokao, involution-anxiety, and Confucian-Daoist cultural milieu. These insights provide a theoretically grounded foundation for developing culturally sensitive interventions to address lying flat among East Asian youth facing intense educational pressure.

### 4.7 Practical implications

This study underscores the critical role of physical exercise in mitigating both behavioral and psychological “lying flat” among college students, with temporal focus and sense of meaning in life serving as key mediators. These findings offer practical strategies for enhancing college students mental well-being and active participation in university settings. First, universities should actively encourage college students to participate in physical exercise by providing accessible programs such as campus sports clubs, fitness classes, or recreational facilities tailored to student schedules. These initiatives can help reduce stress-induced “lying flat” tendency, enabling students to remain active in their academic and social pursuits. Second, universities should implement programs to foster a sense of meaning in life among college students, such as workshops on personal goal-setting, career exploration, or mindfulness practices, to combat the passive mindset associated with psychological “lying flat” and strengthen students motivation. Third, promoting a temporal focus through time management seminars or academic advising sessions can inspire college students to pursue long-term goals, reflect on past experiences, cherish current opportunities, reducing both behavioral and psychological tendencies toward “lying flat”. By adopting these targeted interventions, universities can create a supportive environment that enhances college students mental health and fosters active participation, effectively addressing the “lying flat” phenomenon.

Individuals, particularly college students, can take proactive steps to mitigate the tendency toward “lying flat” by integrating the following practices into their daily lives. Students can aim to engage in moderate physical activities, such as jogging, yoga, or team sports, at least three to four times a week, while setting small, achievable fitness goals can make exercise a sustainable habit. These activities not only reduce stress but also foster positive emotions and improve cognitive function, which can counteract both behavioral and psychological “lying flat”. Second, to combat the passive mindset associated with psychological “lying flat”, students should reflect on their personal values, passions, and long-term aspirations to further clarify their sense of meaning in life. Finally, students can benefit from developing a balanced view of time by setting clear and actionable goals for the future, while appreciating the present and learning from the past.

## 5 Research limitations and prospects

While this study provides valuable insights into the relationship between physical exercise and “lying flat” among college students, it has several limitations. First, the cross-sectional design restricts establishing causal links among physical exercise, temporal focus, sense of meaning in life, and “lying flat” in college students. Longitudinal studies or experimental interventions targeting college students could better clarify these relationships over time. Second, the use of self-reported measures may introduce biases, particularly for psychological “lying flat”, as college students might hesitate to disclose passive attitudes due to social pressures. Incorporating objective measures, such as behavioral observations or physiological stress indicators, could improve the accuracy of findings among this population. Third, our unidimensional operationalization of temporal focus, while parsimonious, overlooks its established multidimensional structure (past, present, future). Future studies should analyze these subscales separately to better understand their unique contributions to “lying flat” and potentially explain non-significant direct effects observed here. Fourth, this study relies on convenience sampling via an online voluntary survey, which may lead to selection bias. Students with higher motivation, greater engagement in physical exercise, or stronger interest in psychological research may have been overrepresented. Although the sample is diverse in terms of university type, major, and grade level, it may not fully represent the general Chinese college student population.

## 6 Conclusion

This study aimed to examine the relationship between physical exercise and the “lying flat” phenomenon among Chinese college students, with a focus on the mediating roles of temporal focus and sense of meaning in life. The findings demonstrated that physical exercise significantly reduces behavioral “lying flat” and indirectly mitigates both behavioral and psychological “lying flat” through the sense of meaning in life, as well as a chain mediation involving temporal focus and sense of meaning in life. Practically, the study advocates college students promoting physical activities, temporal perspectives, and sense of meaning in life programs to address “lying flat”, offering valuable insights for enhancing student mental health and societal positivity.

## Data Availability

The raw data supporting the conclusions of this article will be made available by the authors, without undue reservation.
